# Clinical Factors Associated with Response or Survival after Chemotherapy in Patients with Waldenström Macroglobulinemia in Korea

**DOI:** 10.1155/2014/253243

**Published:** 2014-06-04

**Authors:** Ho Sup Lee, Kihyun Kim, Dok Hyun Yoon, Jin Seok Kim, Soo-Mee Bang, Jeong-Ok Lee, Hyeon Seok Eom, Hyewon Lee, Inho Kim, Won Sik Lee, Sung Hwa Bae, Se Hyung Kim, Mark Hong Lee, Young Rok Do, Jae Hoon Lee, Junshik Hong, Ho-Jin Shin, Ji Hyun Lee, Yeung-Chul Mun, Chang-Ki Min

**Affiliations:** ^1^Department of Internal Medicine, Kosin University College of Medicine, 262 Gamcheon-ro, Seo-gu, Busan 602-702, Republic of Korea; ^2^Department of Internal Medicine, Samsung Medical Center, School of Medicine, Sungkyunkwan University, 81 Irwon-ro, Gangnam-gu, Seoul 135-710, Republic of Korea; ^3^Department of Oncology, Asan Medical Center, University of Ulsan College of Medicine, 13 Gangdong-daero, Songpa-gu, Seoul 138-736, Republic of Korea; ^4^Department of Internal Medicine, Yonsei University College of Medicine, 50-1 Yonsei-ro, Seodaemun-gu, Seoul 120-752, Republic of Korea; ^5^Department of Internal Medicine, Seoul National University Bundang Hospital, 82 Gumi-ro, 173 Beon-gil, Bundang-gu, Seongnam-si, Gyeonggi-do 463-707, Republic of Korea; ^6^Department of Internal Medicine, National Cancer Center of Korea, 323 Ilsan-ro, Ilsandong-gu, Goyang-si, Gyeonggi-do 410-769, Republic of Korea; ^7^Department of Internal Medicine, Seoul National University Hospital, 101 Daehak-ro, Jongno-gu, Seoul 110-744, Republic of Korea; ^8^Department of Internal Medicine, Busan Paik Hospital, 75 Bokji-ro, Busanjin-gu, Busan 614-735, Republic of Korea; ^9^Department of Internal Medicine, Daegu Catholic University Medical Center, 33 Duryugongwon-ro 17-gil, Nam-gu, Daegu 705-718, Republic of Korea; ^10^Department of Internal Medicine, Soonchunhyang University College of Medicine, Bucheon Hospital, 170 Jomaru-ro, Wonmi-gu, Bucheon-si, Gyeonggi-do 420-767, Republic of Korea; ^11^Department of Internal Medicine, Konkuk University Hospital, 120-1 Neungdong-ro, Gwangjin-gu, Seoul 143-729, Republic of Korea; ^12^Department of Internal Medicine, Keimyung University College of Medicine, 56 Dalseong-ro, Jung-gu, Daegu 700-712, Republic of Korea; ^13^Department of Internal Medicine, Gachon University Gil Hospital, 21 Namdong-daero, 774 Beon-gil, Namdong-gu, Incheon 405-760, Republic of Korea; ^14^Department of Internal Medicine, Pusan National University Hospital, 179 Gudeok-ro, Seo-gu, Busan 602-739, Republic of Korea; ^15^Department of Internal Medicine, Dong-A University College of Medicine, 26 Daesingongwon-ro, Seo-gu, Busan 602-715, Republic of Korea; ^16^Department of Internal Medicine, Ewha Womans University School of Medicine, 1071 Anyangcheon-ro, Yangcheon-gu, Seoul 158-710, Republic of Korea; ^17^Division of Hematology, Department of Internal Medicine, Seoul St. Mary's Hospital, College of Medicine, The Catholic University of Korea, 222 Banpo-daero, Seocho-gu, Seoul 137-701, Republic of Korea

## Abstract

Waldenström's macroglobulinemia (WM) is a B-cell proliferative malignancy characterized by immunoglobulin M monoclonal gammopathy and bone marrow infiltration by lymphoplasmacytic cells. Clinical features and cytogenetics of WM in Asia including Republic of Korea remain unclear. Moreover, no study has reported treatment outcomes in patients with WM treated with novel agent combined with conventional chemotherapy. This study investigated clinical features and assessed treatment outcomes with novel agent and conventional chemotherapy in Republic of Korea. Data from all (*n* = 71) patients with newly diagnosed WM at 17 hospitals who received chemotherapy between January 2005 and December 2012 were collected retrospectively. The median age of patients was 66 years (range: 37–92 years) and male to female ratio was 5 : 1. Patients treated with novel agent combined chemotherapy displayed higher overall response rate (ORR) compared to conventional chemotherapy alone (92.9% versus 52.6%, *P* = 0.006). The 5-year overall survival rate was 62.6% (95% confidence interval: 34.73–111.07). Use of novel agents produced higher ORR but survival benefit was not apparent due to the small number of patients and short follow-up duration. Further studies are needed to confirm the efficacy of novel agents in patients with WM.

## 1. Introduction

The consensus group at the Second International Workshop on Waldenström's macroglobulinemia (WM) in 2002 redefined WM as a distinct clinicopathologic entity characterized by bone marrow infiltration by lymphoplasmacytic lymphoma (LPL) and immunoglobulin M (IgM) monoclonal gammopathy [1]. Diagnostic criteria for WM are IgM monoclonal gammopathy of any concentration, bone marrow infiltration by small lymphocytes showing plasmacytoid or plasma cell differentiation, intertrabecular pattern of bone marrow infiltration, and surface IgM^+^, CD5^±^, CD10^−^, CD19^+^, CD20^+^, CD22^+^, CD23^−^, CD25^+^, CD27^+^, FMC7^+^, CD103^−^, and CD138^−^ immunophenotype [2].

WM is a very rare lymphoid malignancy, with an overall incidence estimated at 0.35 for WM and 0.63 for LPL/WM per 100,000 person-years during 2001–2003, representing 1.2% or 2.1% of all non-Hodgkin's lymphomas in the United States Surveillance Epidemiology and End Results (SEER) cancer registries, respectively [3]. Between 1996 and 2003, the crude incidence of LPL/WM was 0.078 per 100,000 person-years in Japan (0.112 for men and 0.048 for women) and 0.032 per 100,000 person-years in Taiwan (0.042 for men and 0.021 for women) [4]. A previous nationwide survey of the incidence of lymphoma based on the REAL classification reported the incidence of LPL in Korea as 0.8%, with the exact incidence rate of WM/LPL not recognized [5]. The incidence rate of WM is lower in the Republic of Korea than those in the USA, which was documented to be about 0.3 per million person-years according to data of the National Cancer Information Center in the Republic of Korea.

The most common clinical manifestations are hepatomegaly (20%), splenomegaly (15%), and lymphadenopathy (15%) [6]. The most common presenting symptom is fatigue related to normochromic or normocytic anemia. The median hemoglobin value at diagnosis is 10 g/dL [7]. Patients with a disease-related hemoglobin level <10 g/dL, platelet count <100 × 10^9^/L, bulky adenopathy or organomegaly, symptomatic hyperviscosity, moderate to severe or advancing peripheral neuropathy on the basis of disease, symptomatic amyloidosis, cryoglobulinemia, or cold-agglutinin disease should be considered for therapy but asymptomatic patients should be observed [8]. Recently, the superior efficacy of chemotherapy combined with novel agent including rituximab, bendamustine, bortezomib, lenalidomide, and thalidomide than that of conventional chemotherapy has been established. However, little is known about the clinical features, epidemiology, and cytogenetics of WM in Asia including the Republic of Korea. Novel agent combined chemotherapy for patients with WM has been restricted in the Republic of Korea because of very low incidence and insurance coverage limitation.

Treatment outcomes in the Republic of Korea patients with WM treated by novel agent combined chemotherapy are unclear. This study is aimed at investigating the clinical features and assess the treatment outcomes of novel agent combined chemotherapy and conventional chemotherapy.

## 2. Materials and Methods

### 2.1. Patients

Data from 71 patients newly diagnosed with WM who received chemotherapy at 17 university hospitals in the Republic of Korea between January 2005 and December 2012 were collected retrospectively. All cases fulfilled the diagnostic criteria [1] and were confirmed as WM by hematopathologists and hematologists. The pretreatment evaluation included a physical examination with performance status evaluation, complete blood cell count with differential count, blood chemistry including lactase dehydrogenase (LDH), protein electrophoresis (PEP), IgM, free light chain kappa and lambda, bone marrow biopsy, chromosomal study, fluorescence in situ hybridization (FISH), and computed tomography (CT) of the chest, abdomen, and pelvis.

### 2.2. Treatment

All patients were treated with conventional chemotherapy or chemotherapy along with novel agent (rituximab, bortezomib, thalidomide, and bendamustine). Bendamustine is old chemotherapeutic agent but recently the roles of this drug were rediscovered by its efficacy and toxicities in indolent lymphoma including WM [9, 10]. Rituximab combined chemotherapy included rituximab, cyclophosphamide, vincristine, and prednisolone (R-CVP) and rituximab, cyclophosphamide, doxorubicin, vincristine, and prednisolone (R-CHOP). Bortezomib combined chemotherapy included bortezomib plus dexamethasone (VD). Thalidomide combined chemotherapy included thalidomide plus dexamethasone (TD) and thalidomide plus cyclophosphamide and dexamethasone (TCD). Bendamustine was used along with prednisolone. Conventional chemotherapy included chlorambucil, CVP, CHOP, melphalan plus prednisolone (MP), cyclophosphamide plus prednisolone (CP), fludarabine plus cyclophosphamide (FC), and fludarabine plus cyclophosphamide and mitoxantrone (FCM). All patients were treated with one or more chemotherapeutic regimens. Patients who displayed progression or intolerance against previous chemotherapy were changed from chemotherapy to a salvage regimen. Introduction of novel agents was applied to four patients as first-line, three as second-line, five at third-line, and two at fourth-line chemotherapy.

### 2.3. Analysis

Overall response rates (ORR) of patients treated with conventional and novel therapy were estimated as the best response at first-line and applied periods. ORR were estimated by clinical parameters in all patients including age, sex, hemoglobin levels, platelet counts, absolute lymphocyte counts (ALC), C-reactive protein (CRP), LDH, serum *β*2-microglobulin, serum albumin, hepatosplenomegaly, Eastern Cooperative Oncology Group (ECOG) performance status, presence of B symptoms, and hyperviscosity syndrome. Those clinical parameters and international staging system (ISS), International Prognostic Scoring System for Waldenstrom's Macroglobulinemia (ISSWM), and treatment modalities were estimated to find prognostic markers for survival. The treatment response was assessed according to the Sixth International Workshop on WM [11].

### 2.4. Statistical Analyses

We investigated independent prognostic factors associated with survival in above clinical and laboratory parameters. The definition of overall survival (OS) was calculated from the date of diagnosis to the date of death from disease-related cause or final follow-up date. Progression-free survival (PFS) was from the date of starting treatment (conventional chemotherapy or novel agent combined chemotherapy) to the date of disease progression, relapse, or death from disease-related cause. Associations between the clinical parameters and ORR were analyzed using the chi-square test. A multiple logistic regression analysis was used for multivariate analysis of independent prognostic factors for ORR. Survival probabilities were calculated according to the Kaplan-Meier method and compared using the log-rank test. The Cox proportional hazards regression model was used for multivariate analysis of independent prognostic factors for survival. Information about the baseline medical status and treatment modalities was collected from the medical records. Approval for these studies was obtained from the Institutional Review Board.

## 3. Results

### 3.1. Clinical and Laboratory Characteristics

The median age of the 71 patients was 66 years (range: 37–92 years) and the male to female ratio was 5 : 1 (Table 1). 25.4% and 38.0% of patients had clinical or radiological evidence of splenomegaly and of lymphadenopathy, respectively. 11.3% of patients had B symptoms before the initiation of treatment. Hyperviscosity and involvement of other organs were shown in 11.3% and 40.8% of patients, respectively. The median serum monoclonal protein level was 3.640 g/dL (range: 0.0183–10.795). The cytogenetic abnormalities identified included deletion of the long arm of chromosome 6 in two cases and absence of trisomy 4. Other cytogenetic abnormalities were identified in 11.3% of cases. Other clinical or laboratory characteristics are summarized in Table 1.

### 3.2. Treatment and Outcomes

The 71 patients were treated with novel agent combined chemotherapy or conventional chemotherapy. 25 patients were treated with chlorambucil with or without prednisone, 14 were treated with CVP or CHOP regimen, 15 were treated with the MP or CP regimen, and three were treated with FC or FCM as first-line therapy. Other patients were treated with novel agent combined chemotherapy. Six patients were treated with R-CVP, R-CHOP, five with VD, two with thalidomide plus dexamethasone, and one with bendamustine plus prednisolone (Table 1). Overall, an objective response (complete or partial response) and more than minimal response (MR) rates were documented in 53.5% and 69.0% of patients, respectively. The median follow-up was 22.97 months. The 5-year PFS and OS rates were 50.5% and 62.6% (95% confidence interval (95% CI): 48.32–81.41 and 34.73–111.07), respectively.

### 3.3. Analysis of Prognostic Factors for Response and Survival

Multiple parameters were analyzed for their possible prognostic impact on ORR and OS. Univariate analysis showed that the following factors were associated with higher ORR (Table 2): higher ALC (<1.0 × 10^9^/L versus ≥1.0 × 10^9^/L; 20.0% versus 64.6%, *P* = 0.069), good ECOG performance status (<2 versus ≥2; 67.4 versus 43.5, *P* = 0.057), and novel agent combined chemotherapy versus conventional chemotherapy (92.9% versus 52.6%, *P* = 0.006). The following factors were associated with superior 5-year OS (Table 2): younger age (<65 years versus ≥65 years; 82.2% versus 36.8%, *P* = 0.024), good ECOG performance status (<2 versus ≥2; 72.6% versus 26.9%, *P* = 0.004), higher serum albumin levels (<3.5 g/dL versus ≥3.5 g/dL; 44.8% versus 84.1%, *P* = 0.010), lower risk international staging system (ISS I, II, and III; 85.7%, 84.8%, and 36.8%, resp.; *P* = 0.004), and novel agent combined chemotherapy versus conventional chemotherapy (100% versus 53.0%, *P* = 0.067). In the multivariate analysis, novel agent combined chemotherapy was an independent prognostic value for ORR (*P* = 0.046) and lower ISS was an independent prognostic value for OS (*P* = 0.008) (Table 3).

## 4. Discussion

The clinical manifestations and laboratory abnormalities associated with WM are related to direct tumor infiltration and to the amount and specific properties of monoclonal IgM. The most common symptoms are weakness and fatigue, usually secondary to anemia. Symptoms of weight loss, excessive sweating, and low-grade fever affect a quarter of patients. Hepatomegaly, splenomegaly, and lymphadenopathy each occur in 15%–30% of patients. Similarly, previous Korean studies reported the most common symptoms as anemia and thrombocytopenia, with other frequently expressed symptoms being (20–40%), hepatosplenomegaly (25–35%), and lymphadenopathy (25–40%) [12–14]. In this study, anemia was the most frequent symptom and thrombocytopenia was secondary frequent symptom with lymphadenopathy, hepatomegaly, and splenomegaly being expressed in 10–40% of the cases. However, hyperviscosity syndrome was documented only in eight cases, which was a relatively low incidence compared to western results [6]. The most common cytogenetic abnormality was deletion of the long arm of chromosome 6 (6q deletion) and trisomy 4 [15, 16]. However, there was only one report about cytogenetics of Korean WM patients, which documented a low rate of 6q deletion (10%) and no trisomy 4 [13]. Similar to a previous Korean study, this study documented a low rate of 6q deletion and other cytogenetic abnormalities.

In this study, novel agent combined chemotherapy was the only independent predictive factor for response rates, although higher ALC count and good performance status were also associated with higher ORR in univariate analysis. Many studies have been shown to improve response and survival rates in patients with WM. Gertz et al. presented meaningful results about efficacy of rituximab in patients with WM [17]. The efficacy of rituximab in WM has been amply described. Rituximab combined chemotherapy including R-CHOP, R-CVP, R-CP, R-F (fludarabine), R-cladribine, and R-CD (cyclophosphamide and dexamethasone) produces superior response rates to conventional chemotherapy [18–23]. Rituximab combined with thalidomide reportedly produced a 72% response rate, and rituximab combined with lenalidomide produced a 50% response rate [24, 25]. Bortezomib has high levels of activity in the management of relapsed WM with response rates ranging from 81% to 96% [26, 27]. In a prospective randomized study of bendamustine plus rituximab compared with R-CHOP in patients with WM, of whom 22 received bendamustine and rituximab and 19 received R-CHOP, the response rate was 95% in both groups, but median progression-free survival was significantly prolonged with bendamustine. The median progression-free survival for R-CHOP was 36 months in contrast to not being reached with bendamustine and rituximab (*P* <  0.0001) [28].

In our study, only lower risk ISS showed superior survival to those of higher risk ISS although younger age, good performance status, higher serum albumin levels, and novel agent combined chemotherapy were associated with longer OS in univariate analysis. Patients treated with novel agent combined chemotherapy especially did not show superior survival rates to conventional chemotherapy in spite of higher ORR in patients receiving novel agents. These results might be associated with small sample size, short follow-up duration, and the clinical features of WM (which seems to be indolent lymphoma). Prior studies have documented several prognostic factors for survival [29–33]. Age, anemia, leukopenia, thrombocytopenia, serum albumin levels, and *β*2-microglobulin values were linked to survival. However, these prognostic factors were not meaningful in this study. Very low incidence rates of WM and restriction of using novel agents because of the limitation of medical reimbursement in Korea might be reasons for the insufficient comparison between novel agent combined chemotherapy and conventional chemotherapy in this study.

## 5. Conclusions

Clinical features of Korean WM are similar to western WM, except for the low incidence of hyperviscosity syndrome. Response rates after chemotherapy were improved by introduction of novel agents such as rituximab, bortezomib, thalidomide, and bendamustine, although survival benefit was not shown. Independent prognostic factor for survival was high risk ISS in Korean WM. However, further study with more patients is needed to determine the efficacy of novel agent combined chemotherapy and to definitively identify the prognostic factors.

## Figures and Tables

**Table 1:** Clinical and laboratory characteristics.

Characteristic	*N* (%) or median (range)
Patients	71
Age, years, median (range)	66 (37–92)
Gender	
Male	59 (83.1)
Female	12 (16.9)
Hemoglobin, g/dL, median (range)	9.6 (3.80–17.10)
Platelet count, ×10^9^/L, median (range)	213 (23–575)
ALC, ×10^9^/L, median (range)	1.70 (0.10–12.30)
CRP, mg/dL, median (range)	2.44 (0.05–23.80)
Serum *β*2-microglobulin, mg/L, median (range)	4.20 (1.34–30.00)
Serum albumin, g/dL, median (range)	3.2 (1.50–4.60)
LDH, IU/L, median (range)	261.0 (74.0–968.0)
BM lymphocyte, %, median (range)	14 (5–100)
Cytogenetic abnormalities, present (%)	8 (11.3%)
Serum monoclonal protein, mg/dL, median (range)	3640.0 (18.30–10795.0)
B symptom, present (%)	8 (11.3)
ECOG (%) ≥2	23 (32.4)
Hyperviscosity, present (%)	8 (11.3)
Lymphadenopathy, yes (%)	27 (38.0)
Extranodal involvement, yes (%)	29 (40.8)
Splenomegaly, yes (%)	18 (25.4)
Hepatomegaly, yes (%)	7 (9.9)
ISS (%)	
I	13 (18.3)
II	26 (36.6)
III	26 (36.6)
Unknown	6 (8.5)
Treatment regimen	
Novel agent combined chemotherapy	14 (19.7)
Conventional chemotherapy	57 (80.3)
Treatment	
Novel group	
R-combined CTx; R-CVP, R-CHOP	6 (8.5)
VD	5 (7.0)
TD	2 (2.8)
Bendamustine plus prednisolone	1 (1.4)
Conventional group	
Chlorambucil	25 (35.2)
CVP or CHOP	14 (19.7)
MP or CP	15 (21.1)
FC or FCM	3 (4.2)

ALC: absolute lymphocyte count; CRP: C-reactive protein; LDH: lactate dehydrogenase; BM: bone marrow; ECOG: Eastern Cooperative Oncology Group performance status; ISS: international staging system; R-combined CTx: rituximab combined chemotherapy; R-CVP: rituximab, cyclophosphamide, vincristine, and prednisolone; R-CHOP: rituximab, cyclophosphamide, doxorubicin, vincristine, and prednisolone; VD: bortezomib plus dexamethasone; TD: thalidomide plus dexamethasone; MP: melphalan plus prednisolone; CP: cyclophosphamide plus prednisolone; FC: fludarabine plus cyclophosphamide; FCM: fludarabine plus cyclophosphamide and mitoxantrone.

**Table 2:** Clinical and laboratory values associated with survival on univariate analysis.

Characteristic	ORR ≥ PR (%)	*P*	5-year PFS (%)	*P*	5-year OS (%)	*P*
Age, years						
<65	66.7	0.327	64.8	0.708	82.2	0.024
≥65	55.3		37.0		36.8	
Gender						
Male	64.4	0.197	46.8	0.530	65.9	0.130
Female	41.7		77.9		45.0	
BM lymphocyte, %						
<50	63.0	0.461	61.8	0.478	40.6	0.610
≥50	52.9		26.2		52.5	
Cytogenetic abnormalities						
Presence	75.0	0.466	64.3	0.599	50.0	0.444
Absence	58.7		50.9		42.4	
Hemoglobin, g/dL						
<11.5	61.3	0.732	54.3	0.140	60.2	0.700
≥11.5	55.6		0.0		87.5	
Platelet count, ×10^9^/L						
<100	69.2	0.479	0.0	0.049	61.9	0.124
≥100	58.6		60.6		63.0	
ALC, ×10^9^/L						
<1.0	20.0	0.069	33.3	0.611	40.0	0.224
≥1.0	64.6		54.3		64.6	
CRP, mg/dL						
<5	58.0	0.817	46.4	0.937	65.5	0.096
≥5	61.5		51.3		48.6	
Serum *β*2-microglobulin, mg/L						
<3	66.7	0.862	48.9	0.130	50.0	0.143
≥3	64.0		50.6		58.4	
Serum albumin, g/dL						
<3.5	58.5	0.683	43.1	0.712	44.8	0.010
≥3.5	63.3		64.4		84.1	
LDH, IU/L						
<450	59.7	0.688	50.1	0.849	61.8	0.403
≥450	66.7		65.6		72.9	
B symptom						
Presence	87.5	0.132	83.3	0.666	60.0	0.385
Absence	56.5		48.8		62.8	
ECOG, (%)						
0-1	67.4	0.057	68.7	0.012	72.6	0.004
≥2	43.5		18.1		26.9	
Hyperviscosity syndrome						
Presence	25.0	0.055	58.3	0.980	0.0	0.918
Absence	63.8		49.7		66.2	
Splenomegaly						
Presence	61.1	0.838	77.4	0.342	0.0	0.300
Absence	58.3		49.5		52.7	
Hepatomegaly						
Presence	57.1	0.884	40.0	0.246	28.6	0.913
Absence	60.0		42.5		45.1	
ISSWM (%)						
Low	57.1	0.567	66.7	0.912	50.0	0.380
Intermediate	58.8		58.7		58.0	
High	75.0		0.0		58.4	
ISS (%)						
I	61.5	0.522	48.7	0.714	85.7	0.004
II	69.2		41.9		84.8	
III	53.8		64.7		36.8	
Treatment regimen						
Novel agent combined chemotherapy	92.9	0.006	79.1	0.418	100.0	0.067
Conventional chemotherapy	52.6		46.3		53.0	

ORR: overall response rates; PR: partial response rates; 5-year PFS: 5-year progression-free survival rates; 5-year OS: 5-year overall survival rates; BM: bone marrow; ALC: absolute lymphocyte count; CRP: C-reactive protein; LDH: lactate dehydrogenase; ECOG: Eastern Cooperative Oncology Group performance status; ISSWM: International Prognostic Scoring System for Waldenstrom's Macroglobulinemia; ISS: international staging system.

**Table 3:** Multivariate analysis for response and survival.

Value	ORR			OS		
	RR	95% CI	*P* value	RR	95% CI	*P* value
Age, years						
<65						
≥65				1.021	0.350–2.980	0.970
ALC, ×10^9^/L						
<1.0						
≥1.0	0.362	0.060–2.193	0.369			
ECOG (%)						
0-1						
≥2	2.006	0.711–5.660	0.188	0.421	0.147–1.208	0.108
Serum albumin, g/dL						
<3.5						
≥3.5				1.123	0.264–4.772	0.875
ISS (%)						
I						
II				0.439	0.078–2.486	0.352
III				0.209	0.066–0.665	0.008
Treatment regimen						
Novel agent combined chemotherapy						
Conventional chemotherapy	5.048	1.032–24.702	0.046	0.368	0.075–1.803	0.217

ORR: overall response rates; OS: overall survival rates; RR: relative risk; 95% CI: 95% confidence interval; ALC: absolute lymphocyte count; ECOG: Eastern Cooperative Oncology Group performance status; ISS: international staging system.
